# Periscope

**Published:** 1882-03

**Authors:** 


### PERISCOPE.

Homœopathic World: November, 1881. Cases are quoted from the *Clinique* showing "the efficacy of a new method for *restoring life to still-born children through the umbilical cord by its mother's breathing*. For this purpose the babe was held by the nurse near to its mother, who was told to take a long breath, and to keep doing so. This being done, the child responded to it, giving, after some seconds, evident signs of returning life, and in less than three minutes it was resuscitated, and cried lustily." The babe's face was also sprinkled with cold water. The above "method" was originally copied from an old *Indian squaw*. Of its practical value, we can say nothing; but its *rationale* is opposed to the teaching of the best obstetricians. After the birth of the child the placenta *should* lay in the cavity of the *uterus detached*, practically a foreign body; hence there is no fœtal circulation, and the mother's heavy breathing could be of no use.

Dr. Burnett quotes from a German work this history of a shoemaker, who had been poisoned with Cicuta virosa: "On my arrival, he was in a terrible state of excitement, singing and yelling, his face very flushed, his eyes glaring and staring about, pupils very much dilated, pulse very rapid." Large doses of *Tart. Stib.* were required to produce emesis. Patient was weak next day and had irritation in throat, ringing in ears. * * * About five weeks after the poisoning he noticed the greater part of the skin of his chest had become *darker* than the rest of his body—became a dark brown color and remained so.

Dr. Berridge relates a case where unvaccinated children after taking *Thuja C.M.* remained in a badly ventilated house with a smallpox patient, during an epidemic, without suffering from the disease Taken from the *Lancet* is a report of a Dr. Gowers, giving several cases of psoriasis caused by long internal exhibition of *Borax. Arsenic* cured it. We quote the following in full (it is too suggestive to lose a word of it), showing as it does the wonderful power of the *simillimum* remedy. *Sanguinaria* can scarcely be considered a tissue remedy.

CASE OF FEARED HARE-LIP: TREATED BY J. C. BURNETT, M. D.

February 18, 1881.—Mrs. ———, æt. twenty-nine, residing in London, has been married four years and a half, and has three children.

FIRST CHILD.—This is a girl, normal in build, but came at the end of the eighth month.

SECOND CHILD.—This is a boy that came at full term, but with single hare-lip of the left side and cleft-jaw.

THIRD CHILD.—Boy at full term, with very slight hare-lip of left side.

*Status præsens:* She believes herself to be in the family way at about the tenth week.

General health of Mrs. ——— and of her husband pretty good. She, herself, tells me that she is subject to headaches in the right temple, and that she has had measles three times. Has a constant feeling of nausea while carrying all her children, and it is *very* bad with this one; it usually lasts about three months. The veins of her hypogastrium were very much dilated with the second child, and slightly so with the first and third. The irritation there from was so great that she was obliged to rise in the night for relief. The veins of her thighs show a good deal. Is subject to piles, and occasionally has prolapse of the rectum. The piles were worse with the second child. She is clearly of a venous diathesis. Her bowels are rather constipated. Her hair is brown.

Her husband has very black hair, and says he at times gets a relaxed throat, and suffers from a torpid liver, for which Dr. Noble occasionally treats him.

These are all the relevant particulars which I was able to obtain from the parents; both very intelligent people.

The father is the product of uncle and niece, but that offered no therapeutic basis. Neither psora, syphilis, nor sycosis seemed present in either of the conjugal pair.

It did not seem to me to be a case of want of the nutritional element, either quantitatively or potentially. The cause seemed to me to lie in the *blood life* of the mother. But where, and in what consisting? That lay beyond my ken; it was, in fact, unknowable.

The essentiality of a state may be unknown and unknowable, but THERE WERE SYMPTOMS IN THE MOTHER, and therefore the scientific application of the law of similars was available. These symptoms were (1) nausea, worse in the evening; (2) sinking at the pit of the stomach before a meal; (3) much salivation; (4) anorexia; (5) aversion to butter. These five symptoms had clearly *some* relationship to the mother's digestive tract, and it is not difficult to suppose that a mother's digestion must necessarily influence the body-fruit within her, both for good and ill. The next question was to determine what proved drug has similar symptoms to those of the mother. I will not make any needless *détour*, but give the drug I diagnosed. It was *Sanguinaria Canadensis.* Take "Allen" and read symptoms: (246) "NAUSEA *in the evening;*" (294) "*SENSATION OF EMPTINESS *in the stomach;*" (244) "Deathly *nausea*, with much SALIVATION;" (230) "*Almost a total* LOSS OF APPETITE;" and (235) "AVERSION TO BUTTER." Thus *Sanguinaria* covered the totality of the symptoms, and it was therefore prescribed. I gave five drops of the third decimal three times a day in a little water.

March 7.—The sinking at the stomach a little better; salivation no better; there is less aversion to butter; appetite much better; naus ea about the same taste bitter; food acid. *Sanguinaria Can.* 6, twenty-four one-drop powders, one night and morning in water.

March 21.—Nausea better; sinking at the stomach better; salivation better; still dislikes butter. The taste is much better, and the food is no longer acid. Has a left-sided headache; is rather constipated; the rectum protrudes a little. *Sanguinaria* 12, given in the same way as last time.

April 4.—Nausea much better, but not quite gone; sinking very much better; still dislikes butter; The headache is gone. "On the whole I am very different from last visit," she said. She thinks the last prescription did her most good. R *Sanguinaria* 30.

April 25.—Nausea still continues a little; the sinking is gone, but it recurs now and again; still does not like butter; salivation nearly gone. R. *Sanguinaria* 1, one pilule three times a day.

June 2.—Nausea gone; she now likes butter; very slight salivation at times, R. *Sanguinaria* 1, to continue taking one pilule at bedtime until the end of the eighth month of utero-gestation.

October 14.—The following letter finishes my story:

"October 10, 1881.

"I have pleasure in giving the particulars you ask for as under:

"1. Born 28th September. 2. Boy (quite perfect). 3. Weight at time of birth, 8 lbs. 4. We expected the arrival about the 15th, so reckon the little one took a fortnight's grace before making his *début.*"

I have nothing to add beyond begging my colleagues to publish their practical experience on this very important and hitherto sadly neglected branch of practical medicine.

LONDON, October 15, 1881.

Dr. F. Ross gives a case illustrating the value of *Urtica Urens* in scalds and burns. He dissolves the $\phi$ in water, and covers the burn with moistened rags, and *keeps them wet.*

December number opens with an editorial on "Crypto-Homœopaths, etc.," which end thus:

"Next comes the twaddling excuse, Oh! I make use of other things besides Homœopathy. The disingenuousness of this is obvious. They know well enough that *every* homœopath makes use of "other things;" he chooses his drugs homœopathically wherever he can, and does everything else which he deems for the advantage of his patients. The real reading of the excuse that they cannot openly own themselves homœopaths because they are not exclusively homœopathic in their views and practice is just this: they only know a *little* Homœopathy, and they prefer every routine palliation to laborious scientific Homœopathy. The genuine homœopath *must* work at his cases and at his *Materia Medica* daily and hourly, and never gets to the end of his studies. There is a quality in Homœopathy like one possessed by the English tongue. Any nigger can speak English in a broken fashion, and so any old woman can do a *little* homœopathic practice; but *all* the English language and *all* Homœopathy is beyond the capacity of the cleverest and most capable living man. No one knows more than a certain portion at any given time—its extent is immensity. The individual who tells us Homœopathy is inadequate for his clinical wants, is like the man who found chess insufficient mental work; is, in fact, simply confessing that he has never really grasped the law and its corrollaries, or he is uttering the truism that some forms of disease are altogether incurable, and therefore Homœopathy does not cure them. God in His wisdom created the shallow waters as well as the deep rivers, and it were vain to expect such a leviathan as is Homœopathy to find anchorage in shallow places. It is not every medico that *can* understand Homœopathy. Our cook deciares that chess is 'a hawful stupid game!'"

A Dr. Ussher writes:

"I don't often use high potencies, for the reason that I get on very well with lower, but as some of our friends at the Convention (London, July, 1881), have labored hard to convince us that these potencies, according to figures, *cannot be* anything, and the use of them has left us more persuaded than ever that we find them useful, will they explain the following case? A patient had five powders of *Sulph.* 200, five globules or more in each, to be taken at intervals of some days. When next she came her story was this: "I have only taken one of the five powders; it vomited me and purged me, leaving my abdomen so sore that I cannot bear to have it touched." "Perhaps," I suggested, "you overtaxed your stomach;" or still willing to accuse the last medicine (as Dr. Dake does), which in this case was *Iodide of Sulph.* 6, a nullity in their eyes; but the answer of the woman was a settler; the vomiting and purging came on immediately after taking the powder. On many occasions this same *Sulph.* 200 has so acquitted itself, and it is so like *Sulphur* that I think there must be *Sulphur* in it, though not exactly enough to make fireworks in one's inside.

January, 1882. Dr. David Wilson writes on "Infinitesimals and the Minimum Dose," quoting Drs. Quain and Russell to prove that Hahnemann's reputation was made by the use of the higher potencies. Dr. Russell, referring to the charge made that "Hahnemann's greatest success was in the first nine years of his practice—i. e. from 1795 to 1804—exclaimed: 'I confess I heard that statement with perfect astonishment.' To me it is perfectly new. I have read all Hahnemann ever published, and I have read and translated about sixty letters written to his most intimate friends. I have read almost all that his early followers have written, and I cannot recall a single expression that warrants such a statement. * * * During the years which he (and Dr. Kidd) fixes as being the most successful of Hahnemann's useful career, he had not proved above *some eight or ten remedies!*"

Dr. Burnett says Cundurango causes "indolent pustules;" "an acne-like eruption;" "colleagues would do well," he says, "to add this little pathogenetic fact to their Materia Medica Pura. A characteristic indication (repeatedly verified), for Cundurango is 'cracks in the corners of the mouth,'" especially when occurring with dyspeptic symptoms.

British Journal of Homœopathy, October, 1881:—In this number appears an extended notice of the Transactions of the "World's Homœopathic Convention, of 1876." The writer, in commenting on an essay by Dr. P. P. Wells on "Eruptive Fevers," says: "When, however, he (Dr. Wells) talks of preventing variola from going on to the suppurative stage by a single dose of *Sulphur*, he seems to forget that such is the usual course of the disease in vaccinated subjects." This sentence is somewhat ambiguous. However, if the writer intended to say, such (*i.e.* suppuration) is *not* the usual course of the disease in vaccinated subjects, we beg leave to differ with him. *Sulphur may* do good in these cases, and also in the unvaccinated subjects.

The writer of this review seems to be a mad critic. For on the same (349) page, he thus pays his respects to Dr. Ad. Lippe. "Dr. Lippe follows with a paper on 'Diphtheria.' He espouses Œrtel's *micrococcus* theory of its propagation, but uses strangely incorrect language in speaking of bacteria as '*vegetable* organisms.'"

We all know Dr. Lippe does not attach much weight to the pathological theories of the day; theories which come and go almost as do the seasons. But when he does mention these theories he generally quotes correctly. We would therefore ask, what is "strangely incorrect" in his speaking of the so-called bacteria as "*vegetable*

organisms." Œrtel, himself, says:* "The most important question in this whole chapter of etiology is that concerning *the relation of certain vegetable organisms to diphtheria.*" Œrtel even speaks of the subject as a "botanical question."

Bacteria were considered by some as synonymous with vibriones, and both were classed as animalcules; *now* we believe both are known to be vegetable organisms. We would not mention these pathological inaccuracies, for we attach little importance to the subject, were they not made by one who pretends to consider *pathology* the *alpha* and *omega* of medical learning.

Had these errors been made by an ignorant *Hahnemannian* we would not have been surprised.

The sentiment "ignorance is bliss where it is folly to be wise," fitly applies to pathology.

January number reiterates the old charge that Hahnemann taught that *Belladonna* was *the* remedy for scarlatina; *Merc. corr.*, for dysentery; *Cinchona*, for intermittents. If the writer really believes these assertions, he had better study Hahnemann more thoroughly.

> "Thou may'st of double ignorance boast,
> Who know'st not that thou nothing know'st."

We have several times reported the very *flattering* notices conferred on The Homœopathic Physician by its journalistic brethren. Had we secured anything but their disapproval, we should have considered it as a sign that we had failed in our work of teaching pure Homœopathy. We take pleasure in quoting (*British Journal of Homœopathy*, Jan., 1882, p. 86), as follows:

"Our readers know the kind of thing [‘*thing*’ is good!] to be expected in such a journal. Four-fifths of each number are occupied with attacks upon the more liberal homœopathists, who are really now doing all the work and all the fighting for the common cause, while the Hahnemannians content themselves with abusing them. We had marked several passages for comment, but think that we can better occupy our space."

Discretion *may* be the better part of valor even to a *Cœur de Lion*. We are glad to hear the "liberal homœopathists are really now doing all the work." We knew they were publishing journals and writing books, but did not know they were doing any *work*.

A verification of the following symptom of *Badiaga* by Dr. C. M.

* Ziemssen, Vol. I, p. 587.

Conant is quoted. " He raises a little, thick, yellowish mucus after a coughing spell, but he seems to have no control over it, for it sometimes flies out of his mouth, half-way across the room." *Badiaga* 30 relieved. *Cannabis Ind.* relieved the following: "Distances seem so long; if I want to go into the next room it seems so far that I feel discouraged before I start."

Dr .J. B .Bell is quoted as aptly saying: " I am reminded of a quotation from a favorite German poet, who in describing a spring, after giving a glowing description of it, says: 'The old man who has viewed its splendors eighty times, stands astonished.' So the man who has a thousand or ten thousand times stood in the presence of a homœopathic cure, stands again astonished."

